# Sodium Succinate as a Corrosion Inhibitor for Carbon Steel Rebars in Simulated Concrete Pore Solution

**DOI:** 10.3390/molecules27248776

**Published:** 2022-12-10

**Authors:** Ahmed Mohamed, Donald P. Visco, David M. Bastidas

**Affiliations:** National Center for Education and Research on Corrosion and Materials Performance, NCERCAMP-UA, Department of Chemical, Biomolecular, and Corrosion Engineering, The University of Akron, 302 E Buchtel Ave, Akron, OH 44325-3906, USA

**Keywords:** corrosion inhibitor, activation energy, quantum chemical DFT, alkaline solution, carbon steel, electrochemical impedance spectroscopy

## Abstract

The inhibiting performance of sodium succinate (Na_2_C_4_H_4_O_4_) was evaluated as an organic environmentally friendly corrosion inhibitor for carbon steel rebars in 0.6 M Cl^−^ simulated concrete pore solution. Potentiodynamic polarization (PDP) and electrochemical impedance spectroscopy (EIS) measurements were utilized to evaluate the inhibitor performance at different temperatures and concentrations. The investigated corrosion inhibitor showed strong corrosion inhibition performance as it adsorbs on the surface of the rebar, creating a protective adsorption film. According to PDP, the inhibitor is classified as a mixed-type inhibitor with an inhibitor efficiency of 77, 69, 59, and 54% for 25, 35, 45, and 55 °C, respectively. EIS validated the PDP tests, showing that sodium succinate displaces the water molecules at the interface, creating an adsorption film by complexing with ferrous ions. The film thickness was calculated, and sodium succinate was able to produce a thicker protective film (span of nanometers) relative to the reference at every temperature. The adsorption of sodium succinate follows the Temkin adsorption isotherm. Δ*G*^0^_ads_ was found to be −32.75 kJ/mol, indicating that the inhibitor adsorption is a combined physisorption and chemisorption process. Different surface characterizations were utilized to substantiate the adsorption of sodium succinate, these include scanning electron microscopy, energy-dispersive X-ray spectroscopy, and micro-Raman spectroscopy. Finally, quantum chemical calculations showed that the delocalized electrons in the carboxyl group have high HOMO energies and electrostatic potential, which facilitates the adsorption of sodium succinate corrosion inhibitor onto the carbon steel rebar surface.

## 1. Introduction

Carbon steel rebars embedded in concrete improve the durability and mechanical performance of concrete structures by increasing its tensile strength [1]. Carbon steel is initially protected against corrosion due to the formation of a passive film promoted by the concrete alkaline environment (pH 12.6), nevertheless, passivity breakdown can occur due to chloride attacks [2]. Concrete structures near marine environments will be exposed to chloride ions that will diffuse into the concrete matrix and initiate an autocatalytic iron acid hydrolysis reaction, as seen in Equation (1) [3]. This reaction will cause the local pH to drop from 12.6 to 4, destabilizing the passive film [4]. As a result, corrosion will initiate and oxyhydroxides, corrosion products, will form on the surface of the rebar. The volume of the rebar will increase, causing stresses that will crack and spall the concrete, leading to a loss of structural integrity [5]. There are many proactive methods used to mitigate the corrosion process in reinforced concrete. These include the use of expensive stainless-steel rebars, coatings, cathodic protection strategies, and the application of organic corrosion inhibitors.

(1) $${\mathrm{Fe}}^{2+}+2{\mathrm{Cl}}^{-}+2H_{2}O\to \;\mathrm{Fe}{\left(\mathrm{OH}\right)}_{2}+2H^{+}+2{\mathrm{Cl}}^{-}$$

Organic inhibitors encompass a wide variety of substances and include amines, alkanolamines, mono-carboxylates, and poly-carboxylates [2]. These organic inhibitors are usually classified as mixed corrosion inhibitors, which decrease the corrosion rate without a significant change in the corrosion potential (*E*_corr_). Organic corrosion inhibitors protect the working electrode by forming an adsorption film that adheres to the surface of the carbon steel rebar through a hydrophilic group, while a hydrophobic group faces the bulk repelling water molecules and protecting the metal surface from the corrosive electrolyte solution [6]. These corrosion inhibitors usually contain polar functional groups with N, S, and O atoms that form five- or six-membered chelate rings due to the bonding between the mentioned functional groups and the metal cation [2].

Among different organic corrosion inhibitors, carboxylic acids and their salts are environmentally friendly and can be derived from fatty acids extracted from vegetable oil, making them an appropriate candidate for corrosion protection in reinforced concrete [7]. These corrosion inhibitors contain a carboxylate group (–COOH) that facilitates the adsorption on the surface of the rebar creating a hydrophobic film through the formation of coordination complexes with ferrous ions [8,9]. The inhibitive properties of carboxylates are attributed to the presence of delocalized electrons, making them nucleophilic reagents for the adsorption process. Additionally, π-bonds, present in the carboxylate group, tend to donate electrons to the metal surface, promoting the corrosion inhibitor adsorption, forming complexes with iron cations, and creating a protective adsorption film [10,11,12].

To illustrate the inhibitive properties of carboxylates, a study investigated the inhibition performance of amines, alkanolamines, and carboxylic acids on carbon steel rebars in 0.1 M Cl^−^ de-aerated SCPS [10]. It was concluded that carboxylic acids had the best inhibitive performance among different organic groups in decreasing the corrosion rate, due to their chelating effect on the surface of the working electrode [10]. Additionally, a quantitative structure–property relationship using Signature molecular descriptors was constructed to illustrate the significance of π-bond electrons (present in the carboxylic groups) in the adsorption process [10]. Moreover, Fazayel et al. studied the corrosion inhibition performance of polycarboxylate derivatives on carbon steel in 0.6 M Cl^−^ SCPS; an inhibition efficiency of 92% was achieved [11]. The standard Gibbs free energy of adsorption (Δ*G*^0^_ads_) was calculated utilizing different adsorption isotherms and the mode of adsorption was found to be a physicochemical adsorption process, due to electrostatic interactions and electron donation between the corrosion inhibitor and the carbon steel sample [11].

Succinic acid, or succinate, is one of the least researched carboxylates as a corrosion inhibitor, especially in reinforced concrete, although it has desirable characteristics that can make it a great candidate to be an environmentally friendly, effective, and economical corrosion inhibitor [13,14]. Succinic acid is a dicarboxylic acid that is soluble in water with two carboxylate groups (–COOH); it is used in many different industries such as medicine, pharmaceuticals, food, and beverages. Succinic acid is also approved by the U.S. Food and Drug Administration, allowing its use as a safe and eco-friendly corrosion inhibitor [14]. One study investigated the anticorrosive properties of succinic acid on carbon steel exposed to produced water of crude oil at different pH: 2, 3, 4, 5, and 6 at 25 °C. It was found that succinic acid was an effective organic corrosion inhibitor at pH ≤ 3 [14]. However, the corrosion inhibition mechanism and quantum chemical properties were not discussed nor studied, especially for reinforced concrete environments. Although, a recent study showed that the addition of succinic acid can increase the compressive strength of concrete, illustrating its compatibility in this environment [15].

The aim of this study is to investigate the corrosion inhibition performance and mechanism of sodium succinate (Na_2_C_4_H_4_O_4_) for carbon steel rebars in 0.6 M Cl^−^ SCPS (pH 12.6) using potentiodynamic polarization (PDP), electrochemical impedance spectroscopy (EIS), and density functional theory (DFT) calculations. Additionally, the effect of temperature and concentration was studied to find the activation energy (*E*_a_), standard Gibbs free energy of adsorption (Δ*G*^0^_ads_), and inhibition mechanism. Moreover, the surface morphology of the carbon steel rebars was investigated by optical and scanning electron microscopy, as well as micro-Raman spectroscopy. Finally, a theoretical quantum chemical computational study was conducted to understand the corrosion inhibition mechanism of sodium succinate on carbon steel.

## 2. Results and Discussion

### 2.1. Electrochemical Results

#### 2.1.1. Potentiodynamic Polarization Curves

**Figure 1:**
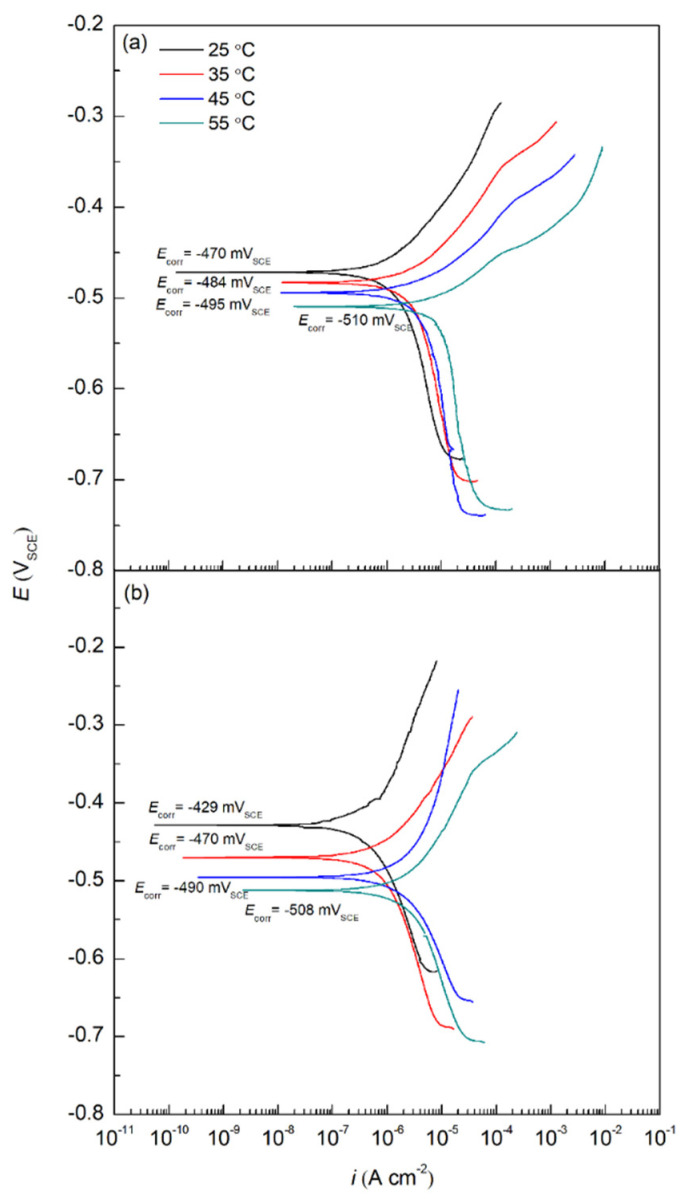
PDP curves for carbon steel rebars in 0.6 M Cl^−^ SCPS in the (**a**) absence, and (**b**) presence of 0.6 M sodium succinate at 25, 35, 45, and 55 °C.

Figure 1 shows potentiodynamic polarization (PDP) curves of carbon steel rebars in 0.6 M Cl^−^ SCPS in the presence and absence of 0.6 M sodium succinate at four different temperatures: 25, 35, 45, and 55 °C. Results obtained from the PDP curves are shown in Table 1 and include *E*_corr_, *i*_corr_, anodic Tafel slope (*β*_a_), and cathodic Tafel slope (*β*_c_). These parameters were determined using Gamry Echem Analyst software, using a potential range of ±20 mV_OCP_. The *IE* for sodium succinate was obtained by using Equation (2):

(2) $$IE\;\left(\%\right)=\left(1-\frac{i_{\mathrm{corr},\mathrm{inh}}}{i_{\mathrm{corr},\mathrm{ref}}}\right)\times 100$$

where *i*_corr,inh_ and *i*_corr,ref_ are the corrosion current density of the inhibited and uninhibited carbon steel rebars (used as a reference), respectively.

**Figure 2:**
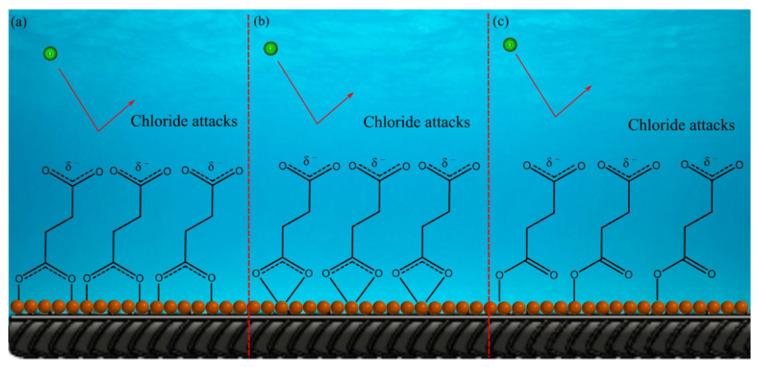
The three main adsorption modes of R–COO– of sodium succinate on the metal surface: (**a**) ή-shape mode, (**b**) bridging mode, and (**c**) chelating mode. Dashed line represents partial π-bonds due to carboxylate resonance. The formation of an adsorption film will repel chloride ions, thus protecting the carbon steel rebar. Ferrous ions are represented as orange spheres.

According to Table 1, the inhibited carbon steel rebars exhibit lower *i*_corr_ values at every temperature indicating a lower corrosion rate. This can be attributed to the adsorption of the sodium succinate on the surface of the carbon steel rebar creating an adsorption film that acts as a barrier between the rebar and the corrosive electrolyte [6,10]. This corrosion protection ability of sodium succinate is clear since the *IE* is 77, 69, 59, and 54% at 25, 35, 45, and 55 °C, respectively. The strong corrosion inhibition performance of sodium succinate can be attributed to the two delocalized negative charged centers found in the succinate anion, helping the inhibitor to easily adsorb on the surface. Once adsorbed the succinate anion will then form complexes with iron cations on the surface of the rebar, creating an adsorption film—protecting the metal surface from the corrosive environment [11,16]. According to Table 1, sodium succinate is classified as a mixed corrosion inhibitor, since the shift in *E*_corr_ is less than 85 mV, indicating that both the metal dissolution (anodic half-reaction) and oxygen reduction reactions (cathodic half-reaction) are being inhibited [17]. Increasing the temperature causes the *i*_corr_ values for the reference and sodium succinate inhibited carbon steel rebars to increase significantly. This is due to accelerated electrochemical kinetics and desorption of the inhibitor from the surface of the working electrode [18,19]. The desorption of the sodium succinate inhibitor will cause areas of the carbon steel rebar to be exposed to the corrosive environment allowing the corrosion process to initiate, thus increasing *i*_corr_. It should be noted that sodium succinate can adsorb on the surface of the rebar and form coordination complexes in different modes. According to Nakamoto, there are three main modes of adsorption between a carboxylate and metal cation; these modes are presented in Figure 2 [8,9].

#### 2.1.2. Electrochemical Impedance Spectroscopy Measurements (EIS)

**Figure 3:**
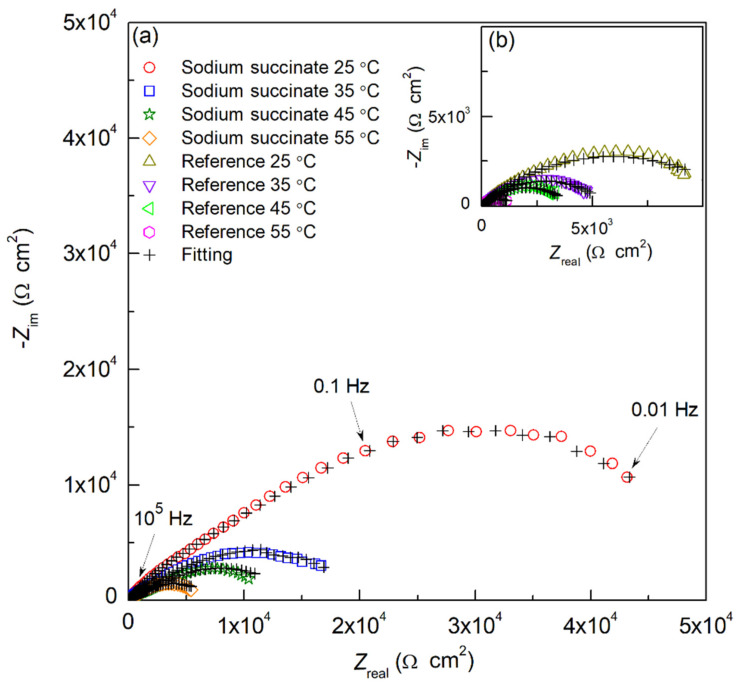
EIS curves for carbon steel rebars in 0.6 M Cl^−^ SCPS in the (**a**) presence, and (**b**) absence of 0.6 M sodium succinate at 25, 35, 45, and 55 °C.

**Figure 4:**
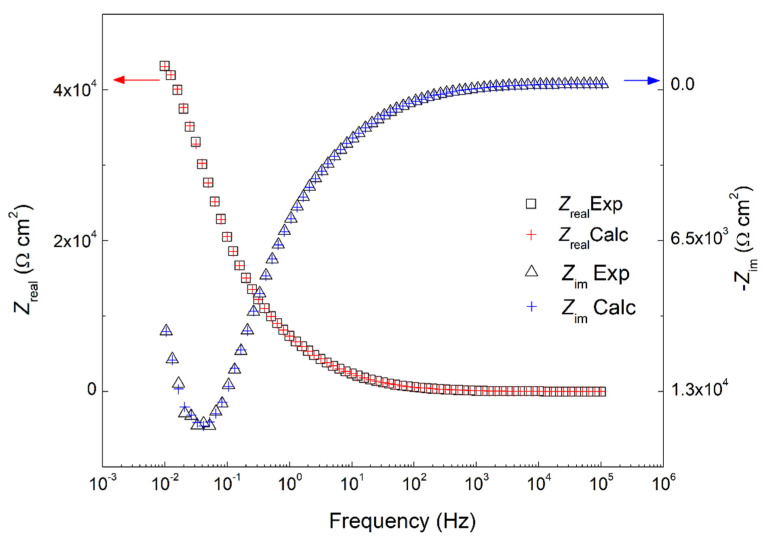
Experimental EIS data of 0.6 M sodium succinate inhibited carbon steel in 0.6 M Cl^−^ SCPS, and calculated values using Kramers–Kronig (KK) transformation at 25 °C.

The electrochemical behavior of carbon steel in the absence and presence of sodium succinate in 0.6 M Cl^−^ SCPS was investigated by the means of EIS at 25, 35, 45, and 55 °C. EIS analysis can confirm the results obtained from the PDP curves and give critical information about the electrochemical double-layer interface between the metal surface and the corrosive electrolyte. Figure 3 illustrates the Nyquist plots of the reference and 0.6 M sodium succinate inhibited carbon steel rebar at the aforementioned temperatures. The Nyquist plots need to be fitted to an electrical equivalent circuit (EEC) to find the required quantitative electrochemical parameters. However, to ensure the robustness of the data obtained before fitting it to an EEC, Kramers–Kronig (KK) transforms were used to evaluate the validity of the experimental results. KK transforms are defined by Equations (3) and (4) [20]:

(3) $$Z_{\mathrm{real}}\left(\omega \right)=Z_{\mathrm{real}}\left(\infty \right)-\left(\frac{2}{\pi }\right)\int_{o}^{\infty }\frac{xZ_{\mathrm{im}}\left(x\right)-\omega Z_{\mathrm{im}}\left(\omega \right)}{x^{2}-\omega^{2}}dx$$

(4) $$Z_{\mathrm{im}}\left(\omega \right)=-\left(\frac{2\omega }{\pi }\right)\int_{o}^{\infty }\frac{Z_{\mathrm{real}}\left(x\right)-Z_{\mathrm{real}}\left(\omega \right)}{x^{2}-\omega^{2}}dx\;$$

where *Z*_real_, *Z*_im_, *ω*, and *x* are the real impedance, imaginary impedance, frequency of the transform, and frequency of the integration, respectively [20]. Comparing the theoretical and the experimental *Z*_real_ and *Z*_im_ can test the robustness of the EIS data obtained. Figure 4 shows a comparison between the experimental EIS data and data obtained from the KK transforms for sodium succinate in 0.6 M Cl^−^ SCPS at 25 °C. As seen in Figure 4, there is a good agreement between the experimental (denoted as symbols) and KK transformed data (denoted as crosses), confirming the robustness of the experiment.

**Figure 5:**
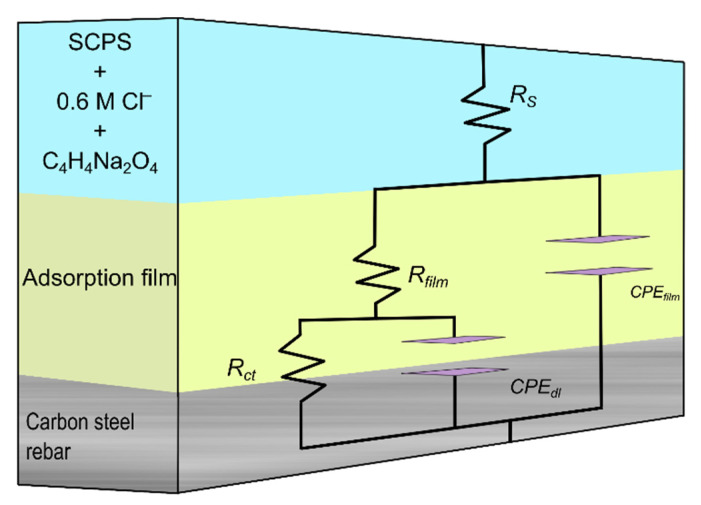
Electrical equivalent circuit (EEC) used to fit EIS data.

The Nyquist plots were fitted to an EEC as shown in Figure 5. The EEC shows different hierarchy-distributed equivalent circuits, where *R*_s_ is the solution resistance, *R*_film_ is the adsorption film resistance, and *R*_ct_ is the charge transfer resistance. Moreover, the EEC is made up of two constant phase elements (*CPE*): one represents the double layer (*R*–*CPE*_dl_) corresponding to low frequencies and the other represents the adsorption film (*R*–*CPE*_film_) corresponding to high frequencies. The effective electrochemical capacitance of the double layer (*C*_eff,dl_), effective capacitance of the passive film (*C*_eff,film_), and effective film thickness (*d*_eff,film_) were calculated using Equations (5)*–*(7) [21,22]:

(5) $$C_{\mathrm{eff},\mathrm{dl}}=Y_{\mathrm{dl}}{}^{\frac{1}{n_{\mathrm{dl}}}}{\left(\frac{1}{R_{s}}-\frac{1}{R_{\mathrm{ct}}}\right)}^{\left(\frac{n_{\mathrm{dl}}-1}{n_{\mathrm{dl}}}\right)}$$

(6) $$C_{\mathrm{eff},\mathrm{film}}=Y_{\mathrm{film}}{\left(\omega_{m}^{\prime \prime }\right)}^{n_{\mathrm{film}\;}-1}$$

(7) $$d_{\mathrm{eff},\mathrm{film}}=\frac{\varepsilon_{o}\;\varepsilon_{\mathrm{film}}}{C_{\mathrm{eff},\mathrm{film}}}$$

where *Y*_dl_ and *Y*_film_ are the admittance of the double layer and film, respectively. Additionally, *n*_film_ and *n*_dl_ are the CPE exponent of the adsorption film and the double layer, respectively, where *n* = 1 indicates an ideal capacitor and *n* = 0 indicates an ideal resistor [23,24]. Finally, *ω*″, *ε*_o_, and *ε* are the frequency where the maximum imaginary impedance is achieved, the vacuum permittivity constant (8.85 × 10^−14^ F cm^−1^), and the dielectric constant of the oxide film (a value of 30 was used [25]), respectively.

As depicted in Figure 3, the Nyquist plots show good agreement between experimental and fitted data. Table 2 summarizes all the values obtained from the EEC fitting for carbon steel rebars in the absence and presence of sodium succinate in 0.6 M Cl^−^ SCPS at 25, 35, 45, and 55 °C. It should be noted that the goodness of the fit (χ^2^) is kept under 10^−3^ and the percentage error of each electrochemical parameter is below 10%.

As seen in Table 2, the *R*_s_ values for the inhibited and uninhibited carbon steel rebars are relatively similar, between 11.35 and 16.03 Ω cm^2^. The *R*_film_ values for the inhibited solution are 6.48 × 10^3^, 4.24 × 10^3^, 3.00 × 10^3^, and 1.80 × 10^3^ Ω cm^2^ compared to the reference’s 2.47 × 10^3^, 1.98 × 10^3^, 1.23 × 10^3^, and 8.46 × 10^3^ Ω cm^2^ at 25, 35, 45, and 55 °C, respectively. The increase in R_f_ in the presence of sodium succinate is due to the formation of a protective adsorption film on the surface of the carbon steel rebar [26,27]. Moreover, the decrease in the film resistance with increased temperature can be attributed to the desorption of the corrosion inhibitor from the surface, hence increased metal dissolution. The *R*_ct_ values for sodium succinate are significantly greater than the reference, indicating lower anodic dissolution kinetics in the presence of the succinate anion. As result, the *IE* was 81.6, 74.3, 65.6, and 59.1% at 25, 35, 45, and 55 °C, corroborating the PDP measurements. The strong anticorrosive performance of sodium succinate is attributed to the formation of different complexes between the succinate anion and the iron cations creating an adsorption film, thus protecting against chloride-induced attacks [6,10,28].

**Figure 6:**
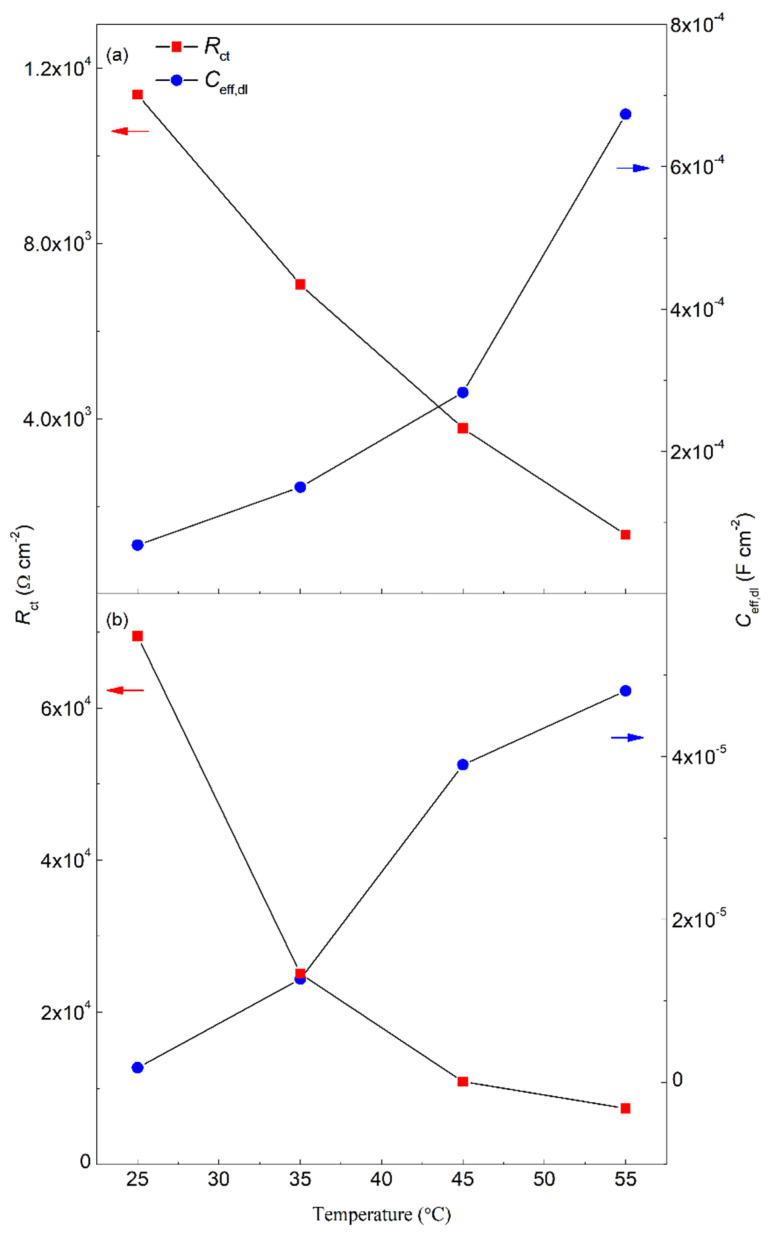
*R*_ct_ and *C*_eff,dl_ of carbon steel in (**a**) absence, and (**b**) presence of 0.6 M sodium succinate in 0.6 M Cl^−^ SCPS at 25, 35, 45, and 55 °C.

**Figure 7:**
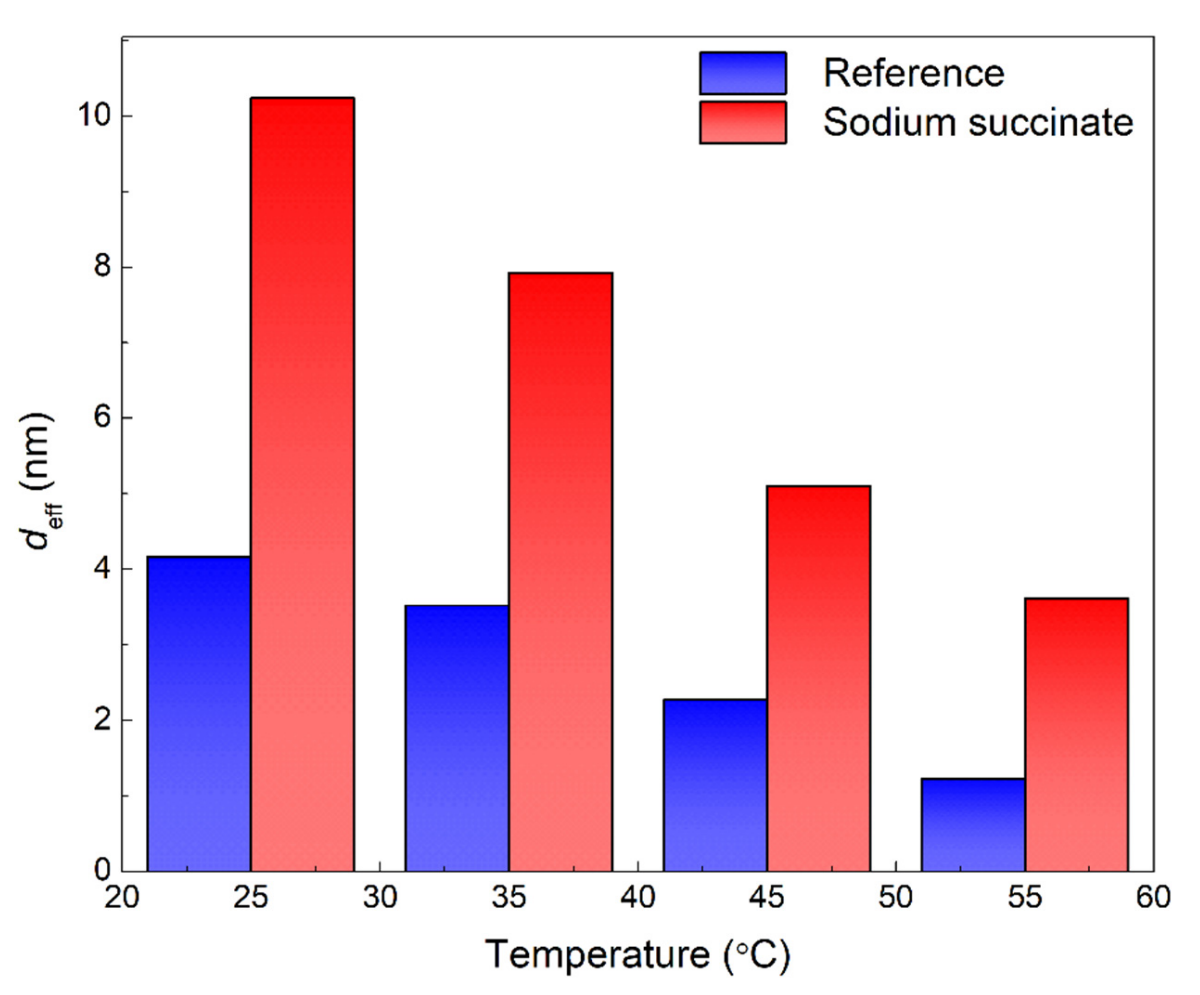
Effective film thickness (*d*_eff,film_) of carbon steel rebars in the presence and absence of 0.6 M sodium succinate in 0.6 M Cl^−^ SCPS at 25, 35, 45, and 55 °C.

The *C*_eff,dl_ of the sodium succinate inhibited carbon steel rebar were lower than the uninhibited (see Table 3), indicating a decrease in the local dielectric constant and/or increase in the thickness of the electrochemical double layer suggesting that the inhibition process is attributed to surface adsorption. Additionally, the lower values of *C*_eff,dl_ are due to the displacement of water molecules at the electrode/electrolyte interface [19,23,29]. The obtained values for *R*_ct_ and *C*_eff,dl_ for carbon steel in the presence and absence of 0.6 M sodium succinate in 0.6 M Cl^−^ SCPS at different temperatures are shown in Figure 6. The variation between the *R*_ct_ and *C*_eff,dl_ are in good agreement, where the highest value of *R*_ct_ corresponds to the lowest value of *C*_eff,dl_—indicating the formation of a protective film [30,31]. The same trend was observed for *C*_eff,film_, lower in the presence of sodium succinate (see Table 3), thus indicating the formation of a thick protective layer. Figure 7 and Table 3 illustrate the *d*_eff_ of the carbon steel rebar in the presence and absence of sodium succinate at different temperatures in 0.6 M Cl^−^ SCPS. Relative to the blank, a thicker film was observed (scale of nanometers) in the presence of sodium succinate corrosion inhibitor, indicating its adsorption and complexation. Nevertheless, at higher temperatures, the film thickness decreased due to the desorption of the corrosion inhibitor.

### 2.2. Activation Thermodynamic Parameters of the Corrosion Process

**Figure 8:**
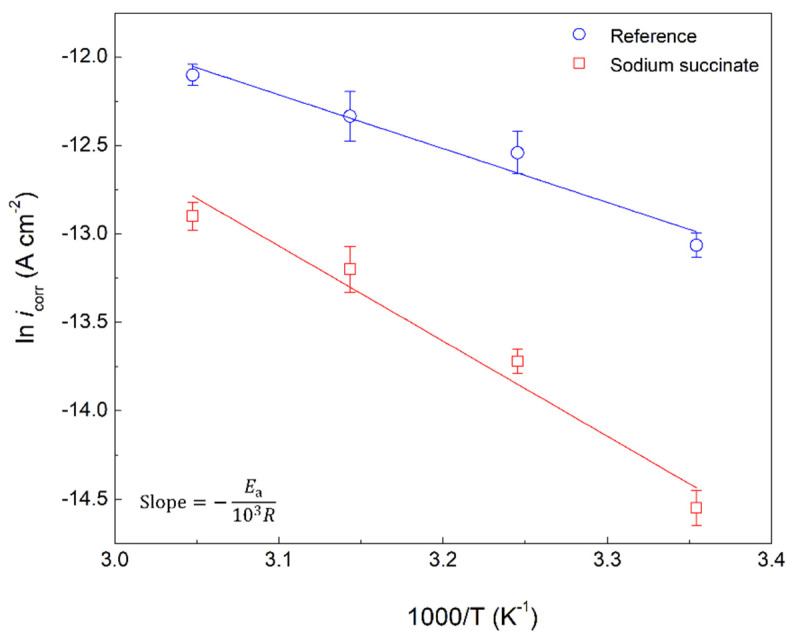
Arrhenius plot of carbon steel rebars in the presence and absence of 0.6 M sodium succinate in 0.6 M Cl^−^ SCPS.

**Figure 9:**
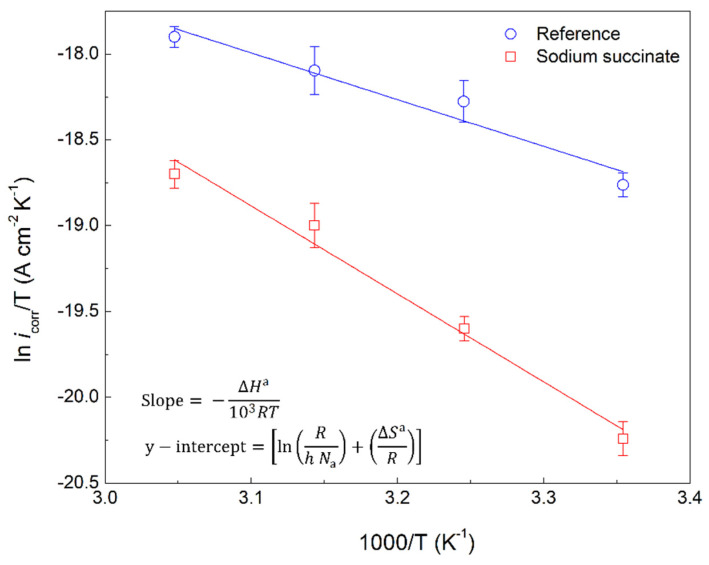
Transition state plot of carbon steel rebars in the presence and absence of 0.6 M sodium succinate in 0.6 Cl^−^ SCPS.

Different thermodynamic parameters can be obtained by testing sodium succinate at different temperatures, these include the activation energy of the corrosion process (*E*_a_), enthalpy of activation (Δ*H*^a^), and entropy of activation (Δ*S*^a^). Equating the forward cathodic and reverse anodic reaction rate at equilibrium will yield a transformed Arrhenius relation enabling the calculation of *E*_a_, as seen in Equation (8) [19,32]:

(8) $$\ln\left(i_{\mathrm{corr}}\right)=\ln\left(A\right)-\frac{E_{a}}{\mathrm{RT}}$$

where *i*_corr_ is the corrosion current density, *A* is the pre-exponential factor, R is the universal gas constant, and T is the temperature. As seen in Figure 8, plotting ln(*i*_corr_) against 1000/T yields a negatively sloped line, where the slope is equivalent to −*E*_a_/10^3^R. Furthermore, using the Eyring transition state will yield Δ*H*^a^ and Δ*S*^a^ for the formation of the activated complex, as seen in Equation (9) [33,34].

(9) $$\ln\left(\frac{i_{\mathrm{corr}}}{T}\right)=\left[\ln\left(\frac{R}{h\;N_{a}}\right)+\left(\frac{\Delta S^{a}}{R}\right)\right]-\frac{\Delta H^{a}}{RT}$$

where Δ*H*^a^, Δ*S*^a^, *h*, and *N*_a_ are the enthalpy of activation, entropy of activation, Planck’s constant, and Avogadro’s number, respectively. As seen in Figure 9, plotting ln(*i*_corr_/T) against 1000/T yields Δ*H*^a^ and Δ*S*^a^ through the slope and y-intercept, respectively. All the activation parameters with the corresponding regression coefficient (R^2^) for the inhibited and uninhibited carbon steel rebar in 0.6 M Cl^−^ SCPS are provided in Table 4. The *E*_a_ increased from 25.35 kJ/mol to 44.73 kJ/mol in the absence and presence of 0.6 M sodium succinate, respectively, indicating a greater energy barrier for corrosion initiation in sodium succinate inhibited solutions. This demonstrates the adsorption of the succinate anion on the surface of the working electrode, blocking the active sites (lowest *E*_a_) and causing the less active (highest *E*_a_) sites to corrode, thus increasing the *E*_a_ of the corrosion process, making it harder to initiate [34,35].

Similarly, Δ*H*^a^ has increased from 22.44 kJ/mol to 42.50 kJ/mol for the reference and sodium succinate, respectively. The positive value of Δ*H*^a^ indicates that the steel dissolution process is an endothermic reaction, therefore a positive correlation between the corrosion rate and temperature [36,37]. It should be noted that Δ*H*^a^ follows the same trend as *E*_a_ and their difference is around 2.6 kJ/mol, which is approximately equal to the average value of RT at the four different temperatures used (see Equation (10)):

(10) $$\mathrm{RT}=E_{a}-\Delta H^{a}\;$$

Hence, the corrosion kinetics satisfy Equation (10) and validate the experimental results, also confirming that the corrosion process is a unimolecular reaction with a single transition state [38,39]. Δ*S*^a^ in the presence of sodium succinate is greater than the reference, signifying increased energy dispersion in the presence of the inhibitor, which is attributed to the displacement of water molecules by the adsorbed succinate anion on the surface of the carbon steel rebar; increased disorder [40]. Moreover, the negative values of the Δ*S*^a^ indicate that the activated complex represents association rather than dissociation [41].

### 2.3. Adsorption Isotherm

**Figure 10:**
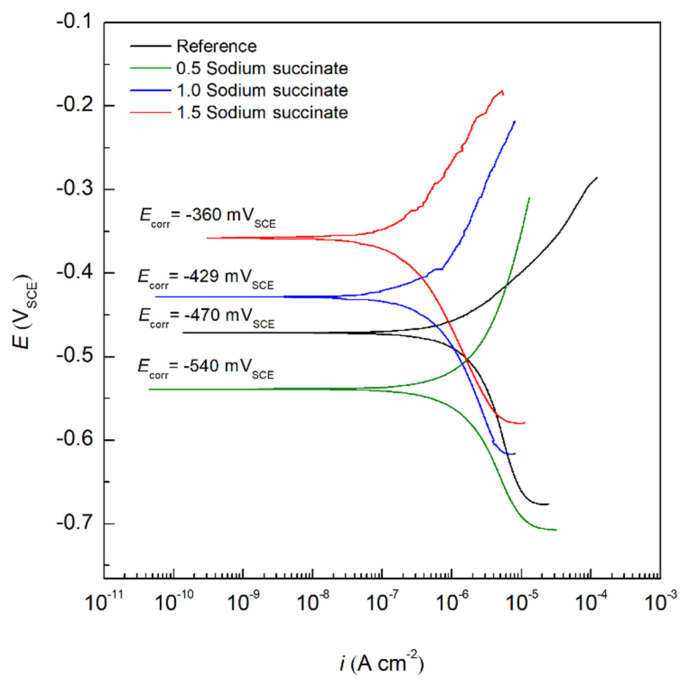
PDP curves of carbon steel submerged in 0.6 M Cl^−^ SCPS in the absence and presence of specific concentrations of sodium succinate at 25 °C.

The corrosion inhibition efficiency of organic molecules, in simple terms, depends on the ability of the inhibitor to adsorb on the surface of the working electrode, thus protecting it from the corrosive environment. In this process, water molecules will be replaced/displaced by the organic corrosion inhibitor creating an adsorption film, as seen in Equation (11) [42]:

(11) $$Org_{\left(\mathrm{sol}\right)}+xH_{2}O_{\left(\mathrm{ads}\right)}\to Org_{\left(\mathrm{ads}\right)}+xH_{2}O_{\left(\mathrm{sol}\right)}$$

where *Org*_(sol)_ is the organic inhibitor in the solution, *Org*_(ads)_ is the adsorbed organic molecule on the metal surface, H_2_O _(ads)_ is the water molecules adsorbed on the surface of the rebar, and *x* is the number of water molecules displaced by the inhibitor. Adsorption isotherms provide information about the interaction of the adsorbed inhibitor and the surface of the carbon steel rebar, which includes the Gibbs free energy of adsorption (Δ*G*^0^_ads_) and the adsorption equilibrium constant (*K*_ads_) [43,44]. In this regard, the effect of sodium succinate concentration was studied, where three different [Na_2_C_4_H_4_O_4_]/[Cl^−^] ratios were tested (0.1, 1, and 1.5) and illustrated in Figure 10. Extrapolating the Tafel slopes yields an *i*_corr_ of 1.2, 0.48, and 0.27 µA cm^−2^ for [Na_2_C_4_H_4_O_4_]/[Cl^−^] ratio of 0.5, 1, and 1.5, respectively. The experimental results indicate a positive correlation between the concentration of sodium succinate and the corrosion inhibition efficiency, since the *IE* is 48, 77, and 87% for 0.5, 1, and 1.5, respectively, at 25 °C in 0.6 M Cl^−^ SPCS. Increased concertation of sodium succinate will ensure the formation of a complete and mature film, preventing the adsorption of Cl^−^ ions.

Table 5 shows all electrochemical parameters for the PDP curves of sodium succinate inhibited and uninhibited carbon steel rebars at the aforementioned [Na_2_C_4_H_4_O_4_]/[Cl^−^] ratios, where the degree of surface coverage (*θ*) and the concentration of corrosion inhibitor (*C*_inh_) were used to test and fit different adsorption isotherms including Langmuir (Equation (12)), Temkin (Equation (13)), and Freundlich (Equation (14)) [29,45]:

(12) $$\frac{C_{\mathrm{inh}}}{\theta }=\frac{1}{K_{\mathrm{ads}}}+C_{\mathrm{inh}}$$

(13) $$e^{\left(-2a\theta \right)}=K_{\mathrm{ads}}C_{\mathrm{inh}}$$

(14) $$\theta =K_{ads}C_{\mathrm{inh}}^{n}$$

where *K*_ads_ is the equilibrium constant of the adsorption reaction and “*a*” describes the molecular interactions in the adsorption layer.

**Figure 11:**
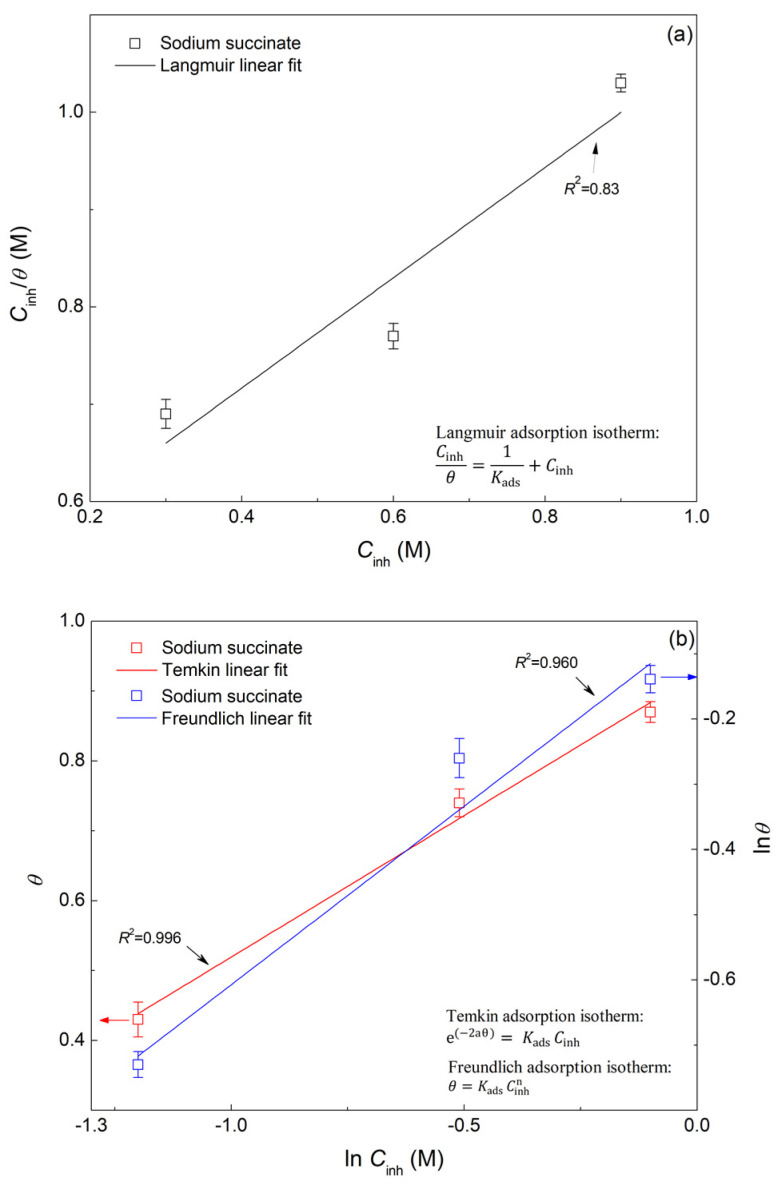
Fittings of different adsorption isotherms for carbon steel in the presence of sodium succinate in 0.6 M Cl^−^ SCPS at 25 °C. (**a**) Langmuir adsorption isotherm, and (**b**) Temkin and Freundlich adsorption isotherms.

It was found that the most accurate fit between the experimental results and the isotherm function, as seen in Figure 11, was achieved by using the Temkin adsorption isotherm which was in accordance with previously published works [14]. Thus, indicating that the adsorption mechanism of sodium succinate obeys the Temkin isotherm having a regression coefficient (R^2^) of 0.996. As a result, by utilizing the slope and *y*-intercept of the fitting in Figure 11, *K*_ads_ was found and Δ*G*^0^_ads_ can be obtained through Equation (15) [46,47]:

(15) $$\Delta G_{\mathrm{ads}}^{0}=-\mathrm{RT}\;\ln\left(55.5\;K_{\mathrm{ads}}\right)$$

where R is the universal gas constant, T is the temperature, and *K*_ads_ is the equilibrium constant of the adsorption/desorption process, and 55.5 is the molar concentration of water [48]. Δ*G*^0^_ads_ values greater than −20 kJ/mol consists of adsorption through electrostatic interaction between the charged metal surface and inhibitor—physisorption. On the other hand, Δ*G*^0^_ads_ values less than −40 kJ/mol involves adsorption on the metal surface through charge transfer between the inhibitor and the metal surface—chemisorption. Moreover, values of Δ*G*^0^_ads_ between −20 kJ/mol and −40 kJ/mol, indicates that the adsorption of the corrosion inhibitor is a combined physisorption and chemisorption process [49]. The experimentally calculated value of Δ*G*^0^_ads_ was found to be −32.75 kJ/mol, indicating that the adsorption of sodium succinate presents a combined contribution of chemisorption and physisorption. The chemical aspect of the adsorption can be attributed to the donation of π-bond electrons in the carboxyl group to the vacant *d* orbitals on the surface of the carbon steel. On the other hand, the physical aspect of the adsorption process is governed by the electrostatic interaction between the charged carboxylate ion and the charged metal surface [11]. Once the inhibitor is adsorbed it will create an adsorption film blocking active sites on the surface of carbon steel rebar, hence creating an energy barrier that will hinder charge transfer, thus decreasing *i*_corr_ and imparting corrosion protection. Finally, the negative value of Δ*G*^0^_ads_ indicates that the adsorption process of sodium succinate on the surface of carbon steel is spontaneous and stable.

### 2.4. Surface Analysis

#### 2.4.1. SEM/EDX Analysis

**Figure 12:**
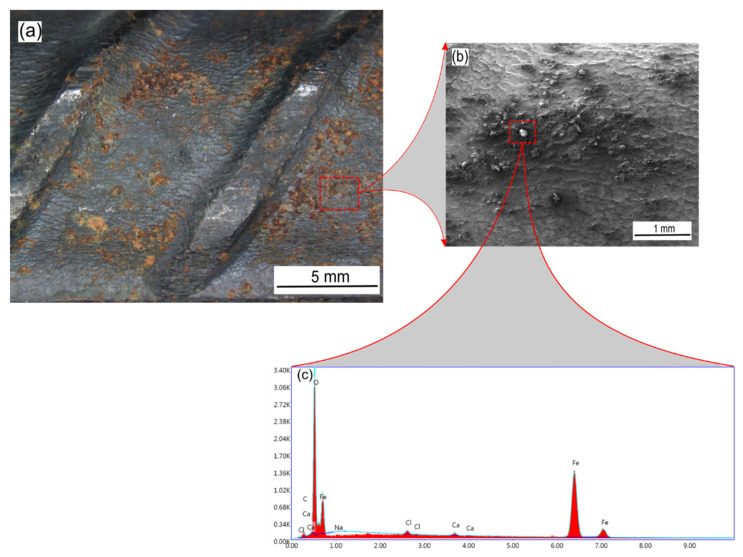
Optical and SEM micrograph of an uninhibited carbon steel rebar in 0.6 M Cl^−^ SCPS at 25 °C. (**a**) Surface of the rebar at ×10, (**b**) surface of the rebar at ×40, and (**c**) EDX elemental spectrum of the surface of the rebar.

The surface morphology of carbon steel rebars in the presence and absence of sodium succinate corrosion inhibitor in 0.6 M Cl^−^ SCPS at 25 °C was studied by the means of an optical microscope and SEM. Figure 12 depicts the surface of the corroded carbon steel rebar in the absence of sodium succinate inhibitor. It is apparent that corrosion has occurred extensively on the surface of the rebar due to chloride induced attacks. Moreover, Figure 12b shows a clear indication of the formation of corrosion products due to the iron dissolution on the uninhibited carbon steel sample, also this can be corroborated using the EDX spectrum (Figure 12c) showing intense oxygen and iron peaks due to the formation of iron oxyhydroxides.

**Figure 13:**
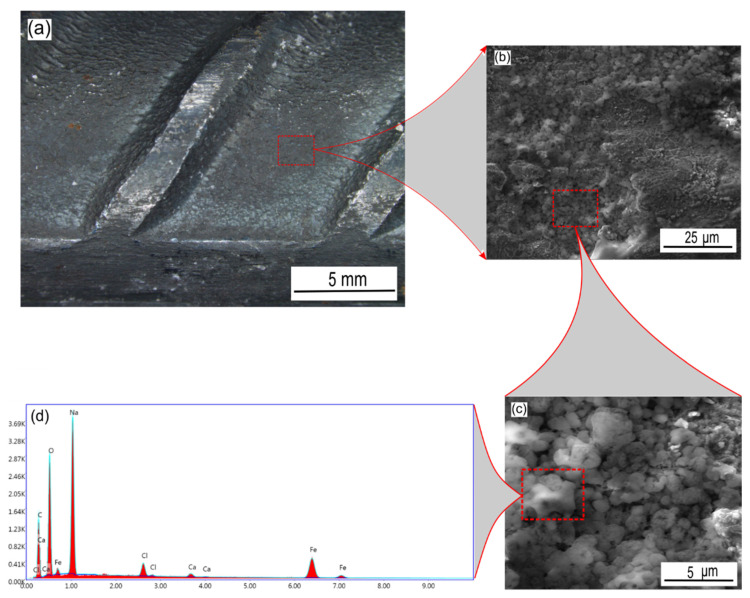
Optical and SEM micrograph of the 0.6 M inhibited carbon steel rebar in 0.6 M Cl^−^ SCPS at 25 °C. (**a**) Surface of the rebar at ×10, (**b**) surface of the rebar at ×2200, (**c**) surface of the rebar at ×8850, and (**d**) EDX elemental spectrum of the complex formation on the surface of the rebar.

Figure 13 represents an SEM micrograph of the surface of carbon steel inhibited by 0.6 M sodium succinate. A clear distinction can be made between the reference and inhibited carbon steel rebar, as almost no corrosion products can be observed on the surface of the working electrode, as seen in Figure 13a. This is attributed to the adsorption of sodium succinate on the surface of the rebar, forming complexes with iron ions and creating an adsorption film that protects the rebar from corrosion [10,11]. These complexes can be observed on the surface in Figure 13c and substantiated by the EDX spectrum presented in Figure 13d. The spectrum shows an abundance of iron, oxygen, carbon, and sodium with a wt.% of 29.72, 19.0, 15.61, and 30.71%, respectively, revealing the presence of complex formation created by the sodium succinate inhibitor consisting of R–COO–Fe [6]. Additionally, chloride ions may be included in the complex formation as R–COO–Cl–Fe explaining their presence in the EDX spectrum [6,50]. It should be noted that the abundance of sodium is due to the presence of sodium atoms in the sodium succinate molecule.

#### 2.4.2. Micro-Raman Spectroscopy

**Figure 14:**
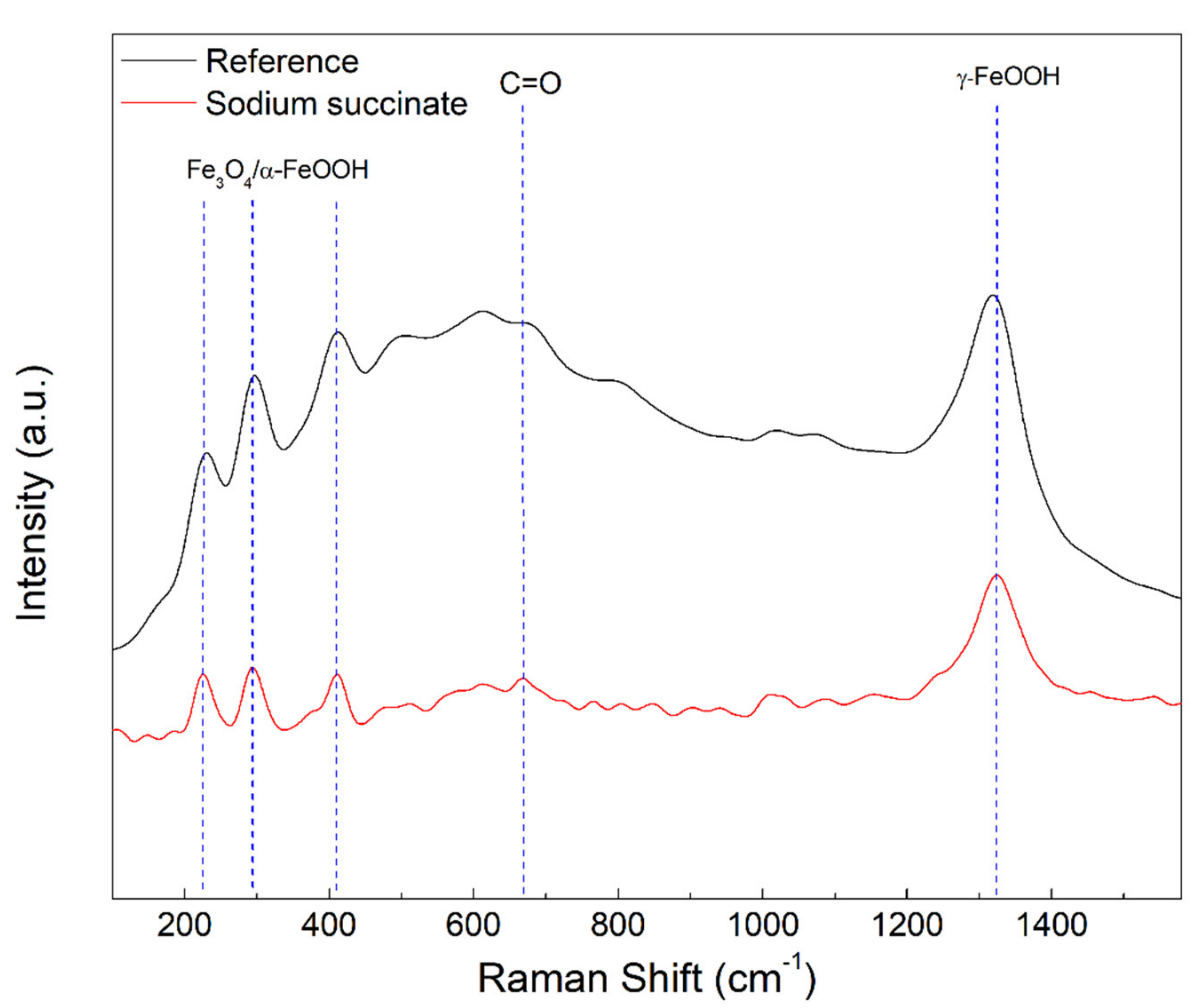
Raman spectroscopy on the inhibited (0.6 M sodium succinate) and uninhibited (reference) rebar surface after exposure to 0.6 M Cl^−^ SCPS at 25 °C.

A micro-Raman spectrometer was used to analyze the composition of the surface of the rebar and further substantiate the formation of the adsorption film. Figure 14 illustrates the Raman spectrum of the reference and 0.6 M sodium succinate inhibited carbon steel in 0.6 M Cl^−^ SCPS at 25 °C. The peaks around 220, 290, 410, and 1320 cm^−1^ are attributed to iron oxides and oxyhydroxides, such as magnetite (Fe_3_O_4_), goethite (α–FeOOH), and lepidocrocite (γ–FeOOH) [51,52]. The peak around 670 cm^−1^ is assigned to C=O bending which can be attributed to the carboxylate groups of the succinate anion [53]. This peak was absent in the uninhibited carbon steel rebar, indicating that sodium succinate was able to adsorb and form a protective film on the surface by complexing with ferrous ions. As a result, the Raman spectrum elucidates the presence of complex formation consisting of iron oxides and sodium succinate corrosion inhibitor.

### 2.5. Quantum Chemical Calculations

**Figure 15:**
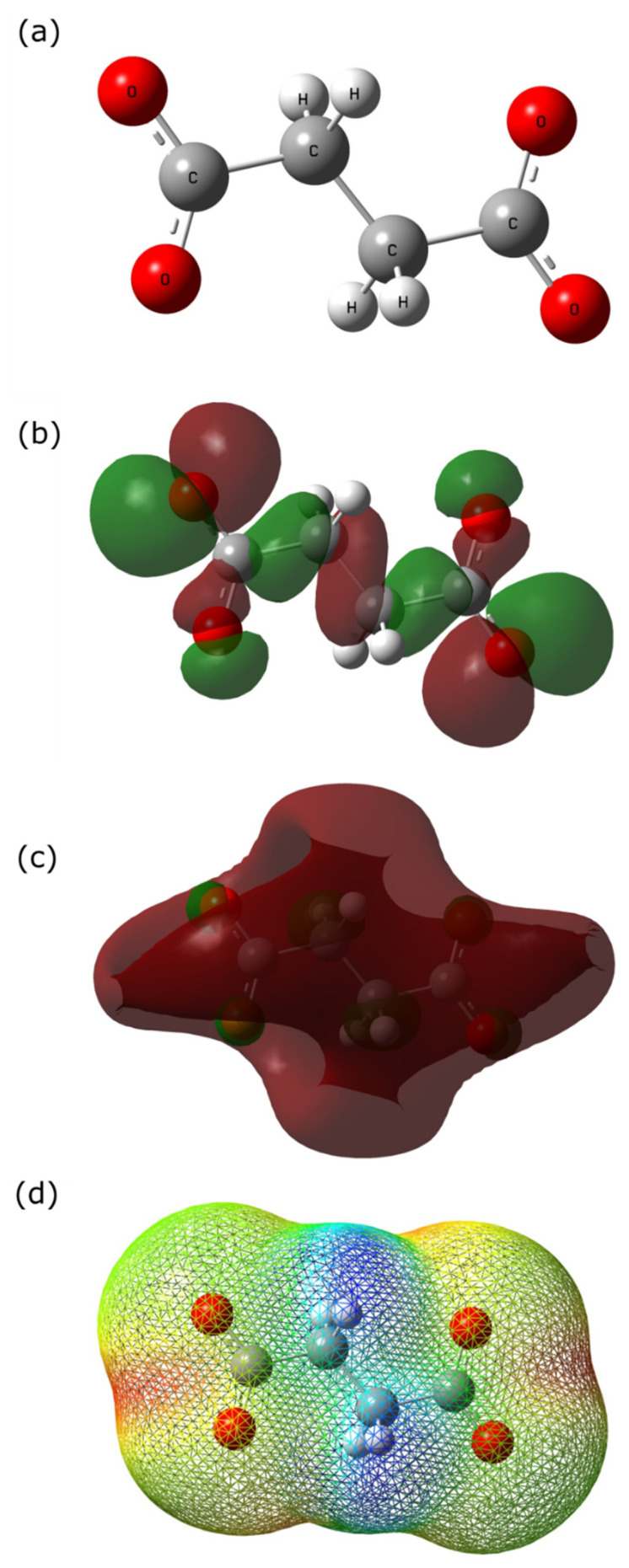
Quantum chemical calculations: (**a**) Optimized molecular structure of succinate anion. Frontier molecular orbital density distribution of succinate anion using DFT: (**b**) HOMO, (**c**) LUMO, and (**d**) electrostatic potential mapping. In (**b**,**c**), green indicates a positive orbital wave function, while red represents a negative orbital wave function. In (**d**) red indicates high electrostatic potentials while blue indicates a low electrostatic potential.

Quantum chemical calculations were used to find a correlation between the inhibitor molecular/electronic structure and its inhibitive properties. The succinate anion molecular geometry was optimized and different quantum chemical parameters were calculated using density functional theory (DFT) method with a Becke’s three-parameter hybrid functional and Lee–Yang–Parr correlation (B3LYP)/6–31G (d,p). Figure 15 represents the optimized molecular structure of the succinate anion followed by the HOMO, LUMO, and electrostatic potential mapping. The calculated quantum chemical data includes the energy of the highest occupied energy molecular orbital (*E*_HOMO_ = −4.64 eV), the energy of the lowest unoccupied molecular orbital (*E*_LUMO_ = 2.37), and the energy gap (Δ*E*_gap_ *= E*_LUMO_ – *E*_HOMO_ = 7.01 eV). Molecules with high *E*_HOMO_ tend to donate electrons to the metal surface compared to molecules with a lower one [54,55]. In contrast, lower *E*_LUMO_ values indicate the ability of a molecule to accept an electron from the metal surface creating a feedback bond [54,56]. The smaller the difference between *E*_HOMO_ and *E*_LUMO_ (i.e., Δ*E*_gap_) determines the kinetic stability, chemical reactivity, and polarizability of a molecule [10,54]. Studies have shown a positive correlation between a low Δ*E*_gap_ and corrosion inhibition, however, it should be noted that the inhibition process is a complex one and many factors can affect it [6,54]. The calculated quantum chemical parameters can be found in Table 6.

**Figure 16:**
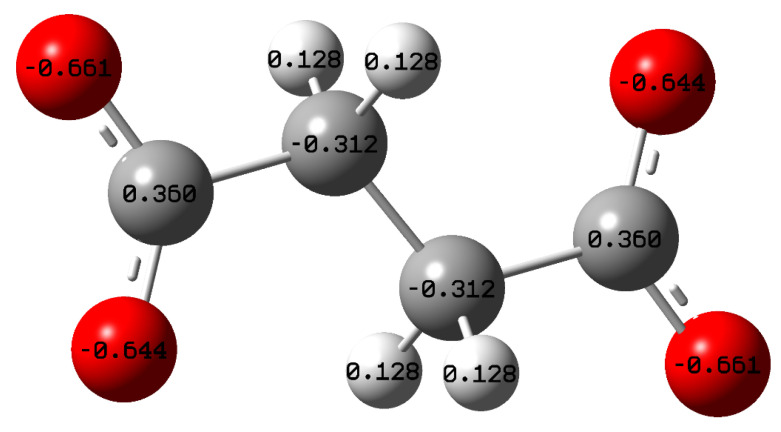
Optimized Geometry of succinate anion with the atomic Mulliken charges using DFT at the B3LYP/6–31 (d, p) basis set level.

According to Figure 15b, HOMO energies are concentrated at the terminal end of the carboxyl group due to the presence of delocalized electrons, indicating the tendency of succinate ion to donate electrons to the unoccupied/vacant *d* orbital of the metal surface. In contrast, the LUMO energies are concentrated on the C–C bonding of the succinate ion, showing that not only succinate donates electrons but also accepts electrons from the metal surface creating a feedback bond, enhancing the adsorption process. Figure 15d shows the electrostatic potential map of the succinate ion, where blue and red colors represent electrophilic and nucleophilic activities, respectively. The red region (most negative potential) of the electrostatic potential map is concentrated between the oxygen atoms at the terminal end of both carboxylic groups. Moreover, the Mulliken charges analysis was performed, as seen in Figure 16, showing that the carboxyl groups hold an excess negative charge indicating that this part of the structure can act as a nucleophilic reagent [36]. Hence, indicating that these positions act as sites for adsorption, where the inhibitor will interact with the metal surface forming complexes with ferrous ions, thus protecting the surface. As a result, sodium succinate corrosion inhibitor is attracted to the steel surface through the electrostatic charge found at the terminal ends of the succinate anion (physisorption), also the adsorption process can occur through sharing electrons from the carboxyl group with unoccupied *d* orbital of the iron surface (chemisorption).

According to Pearson, if two different molecules are brought into proximity of each other, then there will be a charge flow/transfer of electrons from the molecule with low absolute electronegativity to the one with high absolute electronegativity until chemical potential equilibrium is reached [57]. Accordingly, an approximation of the fraction of the electrons transferred (Δ*N*) from the inhibitor to the metallic surface can be calculated using Pearson’s method as seen in Equation (16) [48,57]:

(16) $$\Delta N=\frac{\chi_{\mathrm{Fe}}-\chi_{\mathrm{inh}}}{2\left(\eta_{\mathrm{Fe}}+\eta_{\mathrm{inh}}\right)}$$

where *χ*_Fe_, *χ*_inh_, *η*_Fe_, and *η*_inh_ are the absolute electronegativity of iron, absolute electronegativity of inhibitor, chemical hardness of iron, and chemical hardness of inhibitor, respectively. These parameters are related to the electron affinity (*A*) and ionization potential (*I*_p_), as seen in Equations (17) and (18) [36]:

(17) $$\chi =\frac{\left(I_{p}+A\right)}{2}$$

(18) $$\eta =\frac{\left(I_{p}-A\right)}{2}$$

where *I*_p_ and *A* are related to *E*_HOMO_ and *E*_LUMO_ as seen in Equations (19) and (20) [36]:

(19) $$I_{p}=-E_{\mathrm{HOMO}}$$

(20) $$A=-E_{\mathrm{LUMO}}$$

The *χ*_inh_ and *η*_inh_ parameters were calculated using different values of *I*_p_ and *A* obtained from quantum chemical calculations, while *χ*_Fe_ and *η*_Fe_ were 7 eV and 0 eV for iron, respectively [58]. Δ*N* is a function of global hardness (*η*) and electronegativity (*χ*) for iron and inhibitor, these parameters are calculated and tabulated in Table 6. Finally, the value of Δ*N* > 0 indicates that sodium succinate was the electron donor, while the carbon steel surface was the electron acceptor in the adsorption process [58,59].

## 3. Materials and Methods

Grade 75 carbon steel rebar was used in all electrochemical tests conducted in this study; the composition of the carbon steel rebar can be found in Table 7. The rebar was cut into 4 cm length samples cleaned with ethanol and acetone, then dried with air. The rebar was connected to a copper wire, sealed with red lacquer, and dried for 24 h, with an exposed area of 7.96 cm^2^.

A SCPS was used to simulate the alkalinity of concrete by using a saturated calcium hydroxide solution (Ca(OH)_2_). This solution was filtered and stored at room temperature having a pH of 12.6. The SCPS was contaminated with 0.6 M NaCl to mimic concrete structures exposed to marine environments. Sodium succinate corrosion inhibitor (Na_2_C_4_H_4_O_4_, analytical grade) was added in three different concentrations to have a 0.5, 1.0, and 1.5 molar ratio with Cl^−^. Four different temperatures were used to find different activation parameters: 25, 35, 45, and 55 °C. The pH of the electrolyte solution was measured and maintained at 12.6 after the addition of sodium succinate. It should be noted that sodium succinate will dissociate into succinate anions because of its p*K*_a1_ and p*K*_a2_ values of 4.16 and 5.64, respectively [60,61].

Electrochemical tests were used to evaluate the corrosion inhibition properties of sodium succinate in 0.6 M Cl^−^ SCPS. A three-electrode, temperature-controlled configuration cell was used with a Gamry reference 600 potentiostat. The carbon steel rebar was the working electrode (WE), a saturated calomel electrode (SCE) was the reference electrode (RE), and a platinum mesh was the counter electrode (CE). The open circuit potential (OCP) was monitored until a steady-state *E*_corr_ was achieved. Consequently, electrochemical impedance spectroscopy (EIS) was performed at *E*_corr_ in a frequency range between 10^5^ Hz to 10^−2^ Hz with an applied 10 mV AC excitation signal and a rate of 5 steps/decade, following the ASTM G106-89 standard [62]. Finally, potentiodynamic polarization (PDP) was performed with a scan rate of 0.1667 mV/s from −0.2 V_OCP_ to 0.2 V_OCP_, according to the ASTM G61-86 standard [63]. All tests were performed in triplicates to ensure reproducibility.

The surface analysis of the corroded carbon steel rebars was conducted using a Hitachi–TM3030 scanning electron microscope (SEM) with elemental compositional analysis obtained using energy dispersive X-ray spectroscopy (EDX). A micro-Raman spectrum was obtained using a Horiba LabRam HR micro-Raman spectrometer to elucidate sodium succinate’s adsorption on the rebar’s surface. Finally, quantum chemical calculations were performed using Gaussian 16, where full geometry optimization was carried out on the succinate corrosion inhibitor.

## 4. Conclusions

- Sodium succinate, an environmentally friendly organic compound, possesses physicochemical properties that inhibit corrosion for carbon steel rebars in 0.6 M Cl^−^ contaminated SCPS. Na_2_C_4_H_4_O_4_ creates an organic adsorption film on the surface of the rebar, by forming complexes with ferrous ions that protect the rebar from Cl^−^ induced corrosion.
- The *IE* of Na_2_C_4_H_4_O_4_ according to PDP curves were 77, 69, 59, and 54% at 25, 35, 45, and 55 °C, respectively. The decrease in *IE* with temperature is attributed to the increased corrosion kinetics and desorption of Na_2_C_4_H_4_O_4_ on the surface of the rebar.
- The *IE* of sodium succinate according to EIS were 83.6, 71.2, 65.0, and 59.0% for 25, 35, 45, and 55 °C, respectively, corroborating PDP curves. The *C*_eff,dl_ was calculated at each temperature and was found to be lower than the reference indicating a decrease in the local dielectric constant and/or increase in the thickness of the electrochemical double layer suggesting that the inhibition process is attributed to surface adsorption. The film thickness increased in the presence of sodium succinate at every temperature, due to the formation of R–COO–Fe complexes.
- The activation energy (*E_a_*) is greater in the presence of the inhibitor compared to the reference. This is attributed to the adsorption of the inhibitor on the surface of the carbon steel rebar, making corrosion harder to initiate. Enthalpy of activation (Δ*H^a^*) is positive signifying the endothermic nature of the steel dissolution process. Entropy of activation (Δ*S^a^*) in the presence of the inhibitor is greater than the reference due to disorder from the displacement of water molecules by the adsorbed sodium succinate.
- Sodium succinate follows the Temkin adsorption isotherm. The Δ*G*^0^_ads_ was found to be −32.75 kJ/mol, indicating a combined physicochemical adsorption process.
- Different quantum chemical parameters were calculated to elucidate the experimental results. HOMO energies were found concentrated at the end of the carboxylic group, while LUMO energies were found at the C–C bonds, indicating that the corrosion inhibitor can donate and accept electrons from the metal surface. Finally, the electrostatic potential map shows that the terminal carboxyl groups act as an active site for the adsorption process.

## Figures and Tables

**Table 1:** PDP curves electrochemical parameters of carbon steel in 0.6 M Cl^−^ SCPS in the absence and presence of 0.6 M sodium succinate at 25, 35, 45, and 55 °C.

Sample	Temperature (°C)	*E*_corr_ (mV_SCE_)	*i*_corr_ (µA cm^−2^)	*IE* (%)	*β*_c_ (mV/dec)	*β*_a_ (mV/dec)
Reference	25	–470	2.12	–	288	103
	35	–484	3.56	–	309	77
	45	–495	4.40	–	334	56
	55	–510	5.50	–	445	54
Sodium succinate	25	–429	0.48	77	250	222
	35	–470	1.10	69	275	120
	45	–490	1.80	59	287	127
	55	−508	2.53	54	294	132

**Table 2:** EIS electrochemical parameters of carbon steel in 0.6 M Cl^−^ SCPS in the absence and presence of 0.6 M sodium succinate at 25, 35, 45, and 55 °C.

Sample	Temperature (°C)	*R*_s_ (Ω cm^2^)	*R*_film_ (Ω cm^2^)	*R*_ct_ (Ω cm^2^)	*Y*_film_ (S cm^−2^ s^nf^)	*n*_film_	*Y*_dl_ (S cm^−2^ s^ndl^)	*n*_dl_	*IE* (%)	*χ*^2 (^*^)^
Reference	25	15.66	2.47 × 10^3^	1.28 × 10^4^	3.51 × 10^−6^	0.71	7.21 × 10^−5^	0.71	–	1.16 × 10^−4^
	35	11.49	1.98 × 10^3^	5.62 × 10^3^	5.11 × 10^−6^	0.72	9.14 × 10^−5^	0.73	–	2.51 × 10^−4^
	45	11.97	1.23 × 10^3^	3.96 × 10^3^	7.86 × 10^−6^	0.71	3.95 × 10^−4^	0.73	–	4.44 × 10^−4^
	55	13.94	8.46 × 10^2^	1.37 × 10^3^	1.50 × 10^−5^	0.77	7.84 × 10^−4^	0.78	–	5.25 × 10^−4^
Sodium succinate	25	11.38	6.48 × 10^3^	6.96 × 10^4^	1.11 × 10^−6^	0.74	8.07 × 10^−6^	0.72	81.6	3.60 × 10^−4^
	35	14.36	4.24 × 10^3^	2.19 × 10^4^	1.96 × 10^−6^	0.79	3.42 × 10^−5^	0.80	74.3	3.31 × 10^−4^
	45	17.73	3.00 × 10^3^	1.15 × 10^4^	3.16 × 10^−6^	0.82	8.22 × 10^−5^	0.86	65.6	2.00 × 10^−4^
	55	12.68	1.80 × 10^3^	3.36 × 10^3^	5.13 × 10^−6^	0.85	2.17 × 10^−4^	0.80	59.1	3.58 × 10^−4^

* Total error < 10%.

**Table 3:** *C*_eff,dl_, *C*_eff,film_, and *d*_eff_ of carbon steel rebar in the presence and absence of 0.6 M sodium succinate in 0.6 M Cl^−^ SCPS at 25, 35, 45, and 55 °C.

Sample	Temperature (°C)	*C*_eff,dl_ (F cm^−2^)	*C*_eff,film_ (F cm^−2^)	*d*_eff_ (nm)
Reference	25	4.51 × 10^−6^	6.37 × 10^−6^	4.16
	35	7.24 × 10^−6^	7.53 × 10^−6^	3.52
	45	5.24 × 10^−5^	1.17 × 10^−5^	2.27
	55	2.20 × 10^−4^	2.17 × 10^−5^	1.22
Sodium succinate	25	2.17 × 10^−7^	2.59 × 10^−6^	10.2
	35	5.08 × 10^−6^	3.35 × 10^−6^	7.92
	45	2.84 × 10^−5^	5.20 × 10^−6^	5.10
	55	4.97 × 10^−5^	7.34 × 10^−6^	3.61

**Table 4:** Activation parameters of carbon steel in the absence and presence of 0.6 M sodium succinate in 0.6 M Cl^−^ SCPS.

Sample	R^2^	*E*_a_ (kJ/mol)	Δ*H*^a^ (kJ/mol)	Δ*S*^a^ (J/mol K)	*E*_a_ − Δ*H*^a^ (kJ/mol)
Reference	0.92	25.35	22.44	–276.93	2.91
Sodium succinate	0.95	44.73	42.50	−222.48	2.23

**Table 5:** PDP curves electrochemical parameters for carbon steel in the presence and absence of different concentrations of sodium succinate at 25 °C.

Sample	[Na_2_C_4_H_4_O_4_]/[Cl^−^]	*E*_corr_ (mV_SCE_)	*i*_corr_ (µA cm^−2^)	*IE* (%)	*Θ*	*β*_c_ (mV/dec)	*β*_a_ (mV/dec)
Reference	–	−470	2.12	–	–	288	103
Sodium succinate	0.5	−540	1.20	48	0.48	313	354
	1.0	−429	0.48	77	0.77	250	222
	1.5	−360	0.27	87	0.87	184	149

**Table 6:** Calculated quantum chemical parameters for succinate ion (C_4_H_4_O_4_^2−^).

Quantum Parameter	C_4_H_4_O_4_^2−^
*E*_HOMO_ (eV)	−4.64
*E*_LUMO_ (eV)	2.37
Δ*E*_gap_ (eV)	7.01
*η* (eV)	3.50
*χ* (eV)	1.13
Δ*N*	0.83

**Table 7:** Elemental composition of grade 75 carbon steel rebar (wt.%).

C	Mn	P	S	Si	Cu	Ni	Cr	Mo	V	Fe
0.28	1.08	0.019	0.043	0.20	0.37	0.16	0.16	0.050	0.0379	Bal.

## Data Availability

The raw/processed data required to reproduce these findings cannot be shared at this time as the data also form part of an ongoing study.
